# Triglyceride content increases while cholesterol content decreases in HDL and LDL+IDL fractions following normal meals: the Copenhagen General Population Study of 25,656 individuals

**DOI:** 10.1016/j.atherosclerosis.2023.117316

**Published:** 2023-10

**Authors:** Mia Ø. Johansen, Juan Moreno Vedia, Mie Balling, George Davey Smith, Børge G. Nordestgaard

**Affiliations:** 1 Department of Clinical Biochemistry, Herlev and Gentofte Hospital, Copenhagen University Hospital, Denmark; 2 The Copenhagen General Population Study, Herlev and Gentofte Hospital, Copenhagen University Hospital, Denmark; 3 Institute of Clinical Medicine, Faculty of Health and Medical Sciences, University of Copenhagen, Denmark; 4 Vascular Medicine and Metabolism Unit, Research Unit on Lipids and Atherosclerosis, Sant Joan University Hospital, Universitat Rovira i Virgili, Reus, Spain; 5 Spanish Biomedical Research Centre in Diabetes and Associated Metabolic Disorders (CIBERDEM), Madrid, Spain; 6 MRC Integrative Epidemiology Unit (IEU), Bristol Medical School, University of Bristol, United Kingdom, and; 7 Population Health Sciences, Bristol Medical School, University of Bristol, United Kingdom

## Abstract

**Background and aims:**

During fat tolerance tests, plasma triglycerides increase while high-density lipoprotein (HDL) cholesterol, low-density lipoprotein (LDL) cholesterol, and intermediate-density lipoprotein (IDL) cholesterol decrease. However, it is unknown whether triglyceride content increases and cholesterol content decreases in HDL and LDL+IDL fractions following normal meals in the general population. Therefore, we tested the hypothesis that triglyceride content increases while cholesterol content decreases in HDL and LDL+IDL fractions following normal meals.

**Methods:**

In this cross-sectional study we included 25,656 individuals aged 20–100 years, all without lipid-lowering therapy at examination and selected for metabolomic profiling from the Copenhagen General Population Study. Triglyceride and cholesterol content of 14 lipoprotein fractions were measured using nuclear magnetic resonance (NMR) spectroscopy. Time since last meal was recorded by the examiner immediately before blood sampling.

**Results:**

Following normal meals in age and sex-adjusted analyses and when compared with fasting levels, plasma triglycerides increased for up to 5–6 hours, and triglyceride content increased for up to 6–7 hours in HDL fractions, for up to 6–7 hours in LDL+IDL fractions, and for up to 5–6 hours in very-low-density lipoprotein (VLDL) fractions. Further, plasma cholesterol decreased for up to 1–2 hours, and cholesterol content decreased for up to 2–3 hours in HDL fractions and for up to 5–6 hours in LDL+IDL fractions, while cholesterol content increased for up to 4–5 hours in VLDL fractions.

**Conclusions:**

Following normal meals, triglyceride content increases while cholesterol content decreases in HDL and LDL+IDL fractions.

## Introduction

Previously, lipid profiles were recommended measured in the fasting state, although in most individuals, the postprandial period predominates over the fasting state^1^. However, emerging evidence demonstrates only minimal changes in plasma lipids, lipoproteins, and apolipoproteins following intake of normal meals in the general population^2–4^. In addition, nonfasting plasma triglycerides have been shown to better predict cardiovascular risk than fasting plasma triglycerides^5–7^. Accordingly, recent international lipid guidelines now endorse use of non-fasting lipid profiles for cardiovascular risk prediction^8–10^.

Traditionally, fasting lipid profiles were recommended to limit postprandial increase in plasma triglycerides as observed following oral fat tolerance tests during which individuals typically ingest 1 gram fat per kilogram bodyweight^11^, the latter typically performed in smaller groups of selected individuals during 6–12 hours. In this design, the postprandial increase in plasma triglycerides were often accompanied by a decrease in low-density lipoproteins (LDL) cholesterol and high-density lipoproteins (HDL) cholesterol^12,13^. Whereas most such studies on postprandial lipid profiles evaluate traditional lipid measures including plasma triglycerides, HDL cholesterol, and LDL cholesterol, some studies have additionally reported large postprandial changes in triglyceride and cholesterol content of lipoprotein fractions^12–17^. Notably, in long-term, population-based studies, low HDL cholesterol has been suggested to be a marker of elevated plasma triglycerides and triglyceride-rich remnant lipoproteins^18^. Importantly, it is unknown whether triglyceride content increases while cholesterol content decreases in HDL and LDL+ intermediate-density lipoprotein (IDL) fractions following normal meals in individuals in the general population.

We tested the hypothesis that triglyceride content increases while cholesterol content decreases in HDL and LDL+IDL fractions following normal meals. To test our hypothesis, we cross-sectionally studied 25,656 individuals aged 20–100 years, all without lipid-lowering therapy at examination and selected for metabolomic profiling from the Copenhagen General Population Study. All included individuals had detailed lipid and lipoprotein profiles measured by nuclear magnetic resonance (NMR) spectroscopy of 14 different lipoprotein fractions within HDL, LDL, IDL and very low-density lipoprotein (VLDL).

## Materials and methods

This study was conducted in accordance with the Declaration of Helsinki and was approved by the Danish Data Protection Agency and the Ethics Committee of the Capital Region of Denmark (H-KF-01-144/01). All participants provided written informed consent.

### The Copenhagen General Populations Study

The Copenhagen General Population Study is an ongoing prospective cohort study of 109,751 Danish adults aged 20–100 years, recruited in 2003–2015, and with a participation rate of 43% of those invited. Invited individuals were randomly selected from the national Danish Civil Registration System to reflect the adult white Danish population. At examination, participants filled in a questionnaire, had a physical examination, and had blood samples collected for biochemical analyses. A subgroup of 30,335 individuals were selected for metabolomic profiling using NMR spectroscopy, and among these, we included 25,656 individuals not taking lipid-lowering therapy at examination. Time since last meal (hours) was obtained by the examiner immediately before blood sampling.

### Triglycerides and cholesterol in lipoprotein fractions

High-throughput NMR spectroscopy^19,20^ was used to measure triglyceride content, cholesterol content, and particle number of 14 lipoprotein fractions comprising four fractions of HDL including small (S) HDL, medium (M) HDL, large (L) HDL, and extra-large (XL) HDL; three fractions of LDL including S LDL, M LDL, and L LDL; one fraction of intermediate-density lipoprotein (IDL); and six fractions of VLDL including extra small (XS) VLDL, S VLDL, M VLDL, L VLDL, XL VLDL, and chylomicrons and extra extra-large (XXL) VLDL. To preserve lipoprotein composition during long-term storage, serum samples were stored at -80 □ until NMR analysis. The NMR analyses were conducted using the Nightingale assay at the Metabolomic Core Facility at the University of Bristol.

### Covariates

Weight (kg) and height (m) were measured by the examiner on the day of examination, and body mass index (BMI) was calculated as weight (kg) divided by height squared (m^2^). Plasma cholesterol, plasma triglycerides, and plasma albumin were measured on fresh blood samples at the day of examination using validated standard biochemical assays at the Department of Clinical Biochemistry at Copenhagen University Hospital in Herlev.

### Statistical analyses

Stata/SE 17.0 was used to perform all statistical analyses. Missing information (0.49% of all covariate information) were imputed based on age and sex using single imputation; results were similar without imputation. As is a standard procedure in ultracentrifugation^21^ and previously done using NMR spectroscopy^22–24^, triglyceride and cholesterol content of lipoprotein fractions were corrected for recovery relative to plasma triglycerides and plasma cholesterol measured on fresh blood samples at the day of examination to obtain clinically relatable lipid levels. Distribution of plasma triglycerides, triglyceride content in all lipoprotein fractions, cholesterol content in VLDL fractions, and VLDL particle number were skewed and therefore logarithmically transformed in statistical analyses to approach normal distribution.

General linear regression models were used to adjust for age and sex; for age; and for age, sex, and plasma albumin. Student *t*-tests compared between groups plasma triglycerides, plasma cholesterol, triglyceride content in lipoprotein fractions, cholesterol content in lipoprotein fractions, and particle number according to time since last meal ranging from fasting (≥8 hours), 0–1 hour, 1–2 hours, 2–3 hours, 3–4 hours, 4–5 hours, 5–6 hours, 6–7 hours, or 7–8 hours with fasting (≥8 hours) as the reference. For sensitivity, we added analyses stratified according to BMI and sex, and analyses additionally adjusted for plasma albumin to account for haemodilution following intake of fluid.

In analyses stratified according to BMI, individuals were divided into two groups based on the World Health Organization (WHO) classification of overweight (BMI≥25kg/m^2^); one group with BMI <25 kg/m^2^ and another group with BMI≥25kg/m^2^. To account for multiple comparisons, we used a Bonferroni corrected p-value of < 0.05/8 = 0.00625.

## Results

Among 30,335 individuals selected for metabolomic profiling nested within 109,751 individuals from the Copenhagen General Population Study, we studied 25,656 individuals aged 20–100 years and without lipid-lowering therapy at examination (Supplementary Figure 1). Baseline characteristics according to time since last meal are shown in Table 1.

### Triglycerides and cholesterol in plasma and main lipoprotein fractions

**Figure 1:**
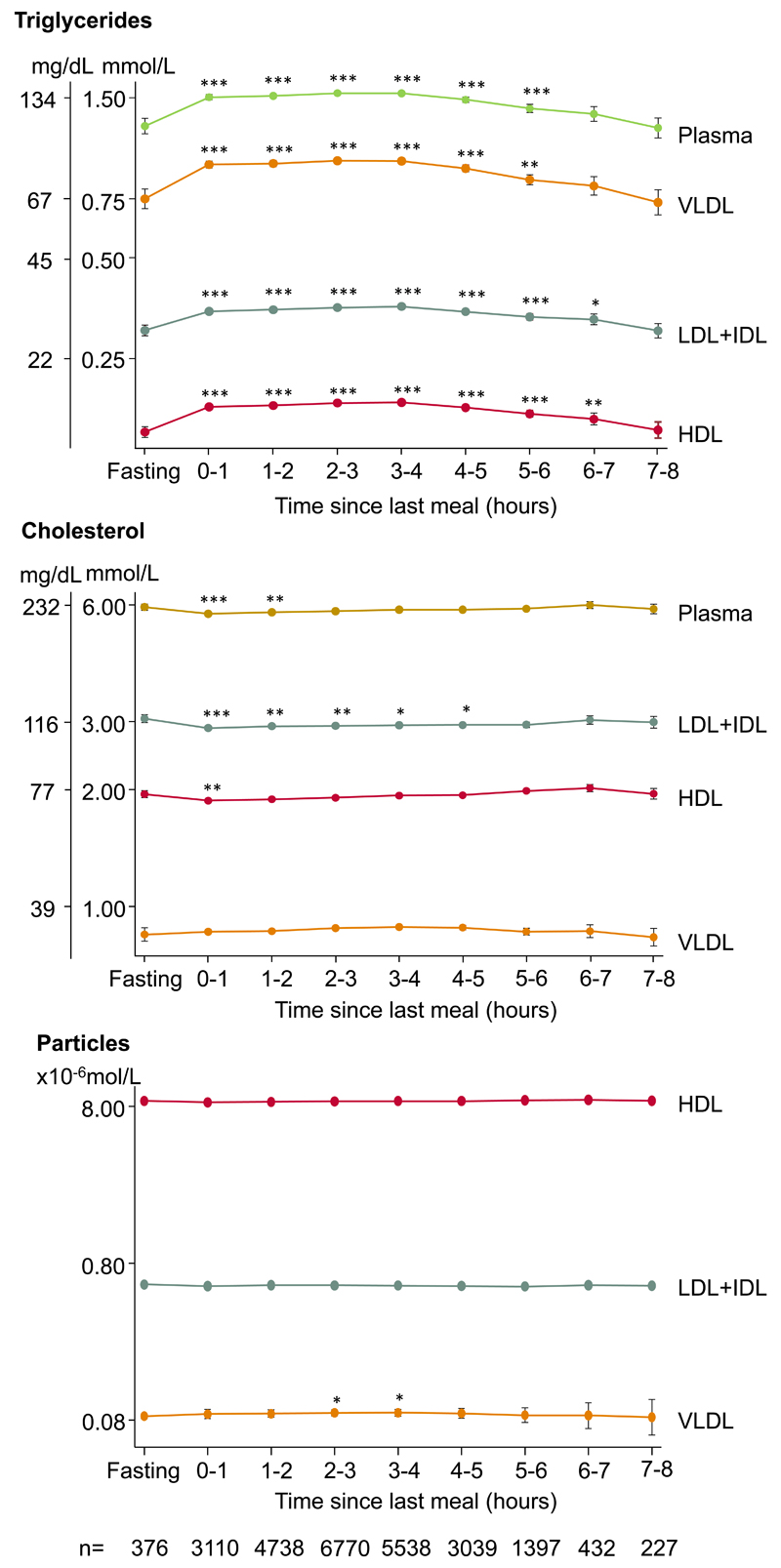
Triglycerides, cholesterol, and lipoprotein particle number in plasma and in main lipoproteins fractions according to time since the last meal. Dots represent quadratic means adjusted for age and sex and error bars represent 95% confidence intervals. Plasma triglycerides and plasma cholesterol were measured on fresh blood samples using routine hospital assays. Triglyceride and cholesterol content in lipoprotein fractions were measured using nuclear magnetic resonance spectroscopy and corrected for recovery. The y-axes are on a logarithmic scale. Bonferroni corrected *p*-values based on 8 parallel tests using unpaired Student *t*-test versus fasting levels (≥8 hours) are as follows: *p<0.05; **p<0.01; ***p<0.001. Abbreviations: HDL: high-density lipoproteins. IDL: intermediate-density lipoproteins. LDL: low-density lipoproteins. VLDL: very low-density lipoproteins.

In age and sex-adjusted analyses and when compared with fasting levels, HDL triglycerides increased for up to 6–7 hours, LDL+IDL triglycerides increased for up to 6–7 hours, VLDL triglycerides increased for up to 5–6 hours, and plasma triglycerides increased for up to 5–6 hours (Figure 1, upper panel). Further, HDL cholesterol decreased for up to 0–1 hour, LDL+IDL cholesterol decreased for up to 4–5 hours, and plasma cholesterol decreased for up to 1–2 hours, while no changes in VLDL cholesterol were observed (Figure 1, middle panel). When compared with fasting levels, VLDL particle number increased for up to 3–4 hours (Figure 1, bottom panel). Results were similar in women and men, although most pronounced in men (Supplementary Figure 2).

A meal often is accompanied by fluid intake resulting in haemodilution, and therefore we added analyses additionally adjusted for plasma albumin (Supplementary Figure 3). In these analyses, plasma cholesterol, HDL cholesterol, and LDL+IDL cholesterol did no longer vary according to time since last meal, while the observed increase in plasma triglycerides, triglyceride content of lipoprotein fractions, and VLDL particle number remained.

### Triglycerides in lipoprotein fractions

**Figure 2:**
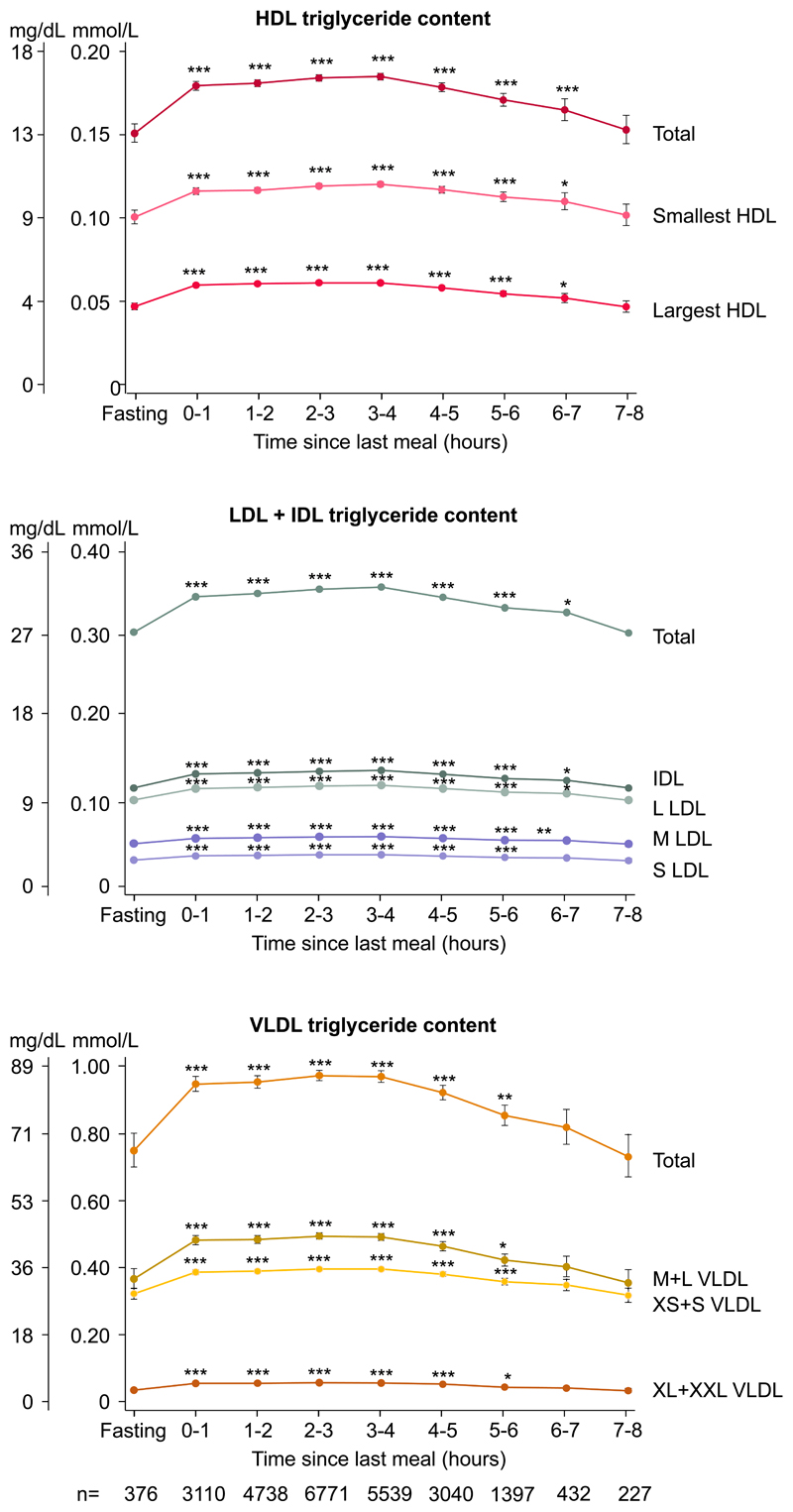
Concentrations of triglycerides in lipoproteins fractions according to time since last meal. Dots represent quadratic means adjusted for age and sex and error bars represent 95% confidence intervals. Triglyceride content in lipoprotein fractions were measured using nuclear magnetic resonance spectroscopy and were corrected for recovery. Bonferroni corrected *p*-values based on 8 parallel tests using unpaired Student *t*-test versus fasting levels (≥8 hours) are as follows: *p<0.05; **p<0.01; ***p<0.001. Smallest HDL include S+M HDL. Largest HDL include L+XL HDL. Abbreviations: HDL: high-density lipoproteins. IDL: intermediate-density lipoproteins. L: large. LDL: low-density lipoproteins. M: medium. S: small. VLDL: very low-density lipoproteins. XL: extra large. XS: extra small. XXL: extra extra large.

In age and sex-adjusted analyses and when compared with fasting levels, triglyceride content increased for up to 6–7 hours in both the smallest and largest HDL fractions, for up to 5–6 hours in S LDL, for up to 6–7 hours in M LDL, L LDL, and IDL, and for up to 5–6 hours after the last meal in all VLDL fractions (Figure 2).

### Cholesterol in main lipoprotein fractions according to plasma triglycerides

**Figure 3:**
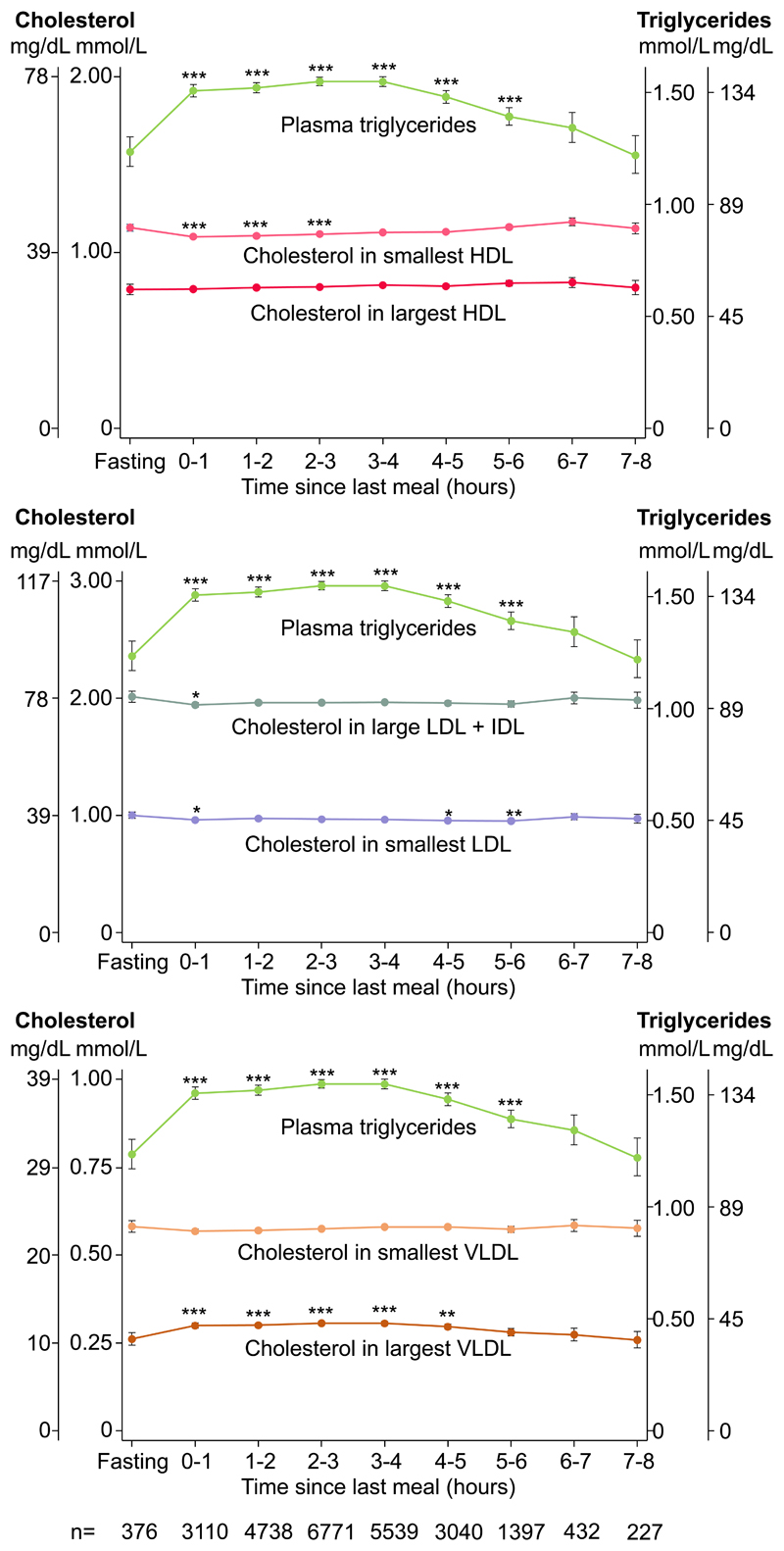
Cholesterol in main lipoprotein fractions along with plasma triglycerides according to time since last meal. Dots represent quadratic means adjusted for age and sex and error bars represent 95% confidence intervals. Plasma triglycerides were measured on fresh blood samples using routine hospital assays. Cholesterol content in lipoprotein fractions was measured using nuclear magnetic resonance spectroscopy and was corrected for recovery. Left y-axis shows lipoprotein cholesterol concentration; right y-axis shows plasma triglyceride concentration. Bonferroni corrected *p*-values based on 8 parallel tests using unpaired Student *t*-test versus fasting levels (≥8 hours) are as follows: *p<0.05; **p<0.01; ***p<0.001. Smallest HDL include S+M HDL. Largest HDL include L+XL HDL. Smallest LDL include S+ M LDL. Smallest VLDL include XS+ S VLDL. Largest VLDL include M+ L+XL+ XXL VLDL. Abbreviations: HDL: high-density lipoproteins. IDL: intermediate-density lipoproteins. L: large. LDL: low-density lipoproteins. M: medium. S: small. VLDL: very low-density lipoproteins. XL: extra large. XS: extra small. XXL: extra extra large.

In age, sex, and plasma albumin-adjusted analyses, the observed increase in plasma triglycerides for up to 5–6 hours following the last meal was accompanied by decreased cholesterol content in the smallest HDL fractions for up to 2–3 hours and in LDL+IDL fractions for 0–1 hour, and increased cholesterol content in the four largest fractions of VLDL for up to 4–5 hours after last meal (Figure 3). In contrast, cholesterol content in the two largest subgroups of HDL and in the two smallest subgroups of VLDL remained unaltered.

### Stratification by body mass index

**Figure 4:**
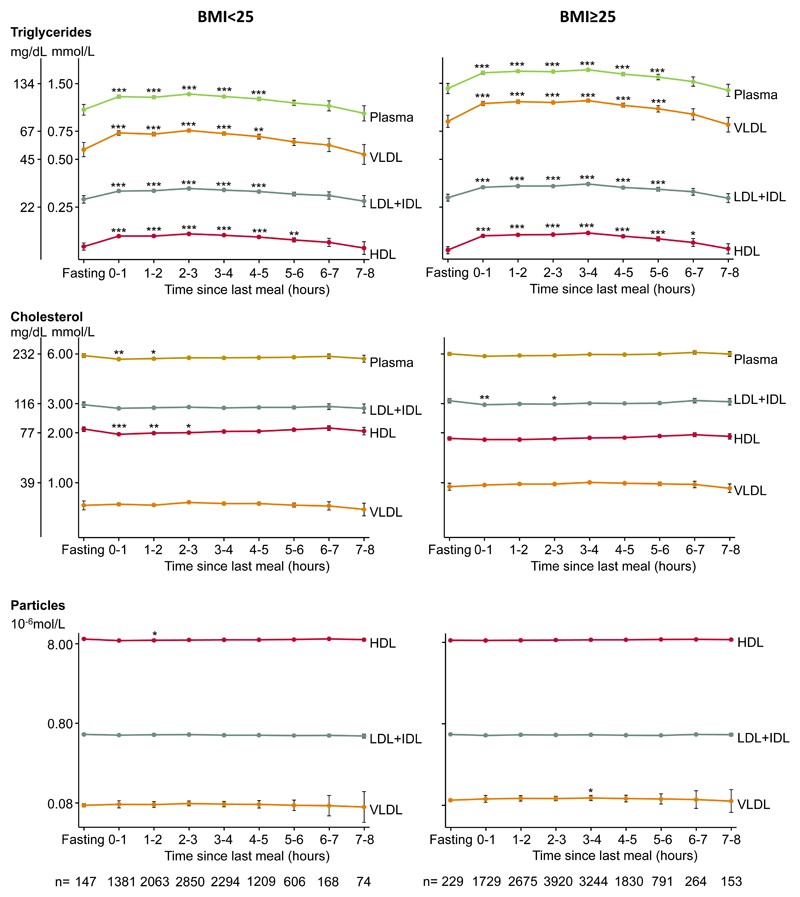
Triglycerides, cholesterol, and lipoprotein particle number in plasma and in main lipoproteins fractions according to time since the last meal in individuals with normal weight and overweight/obesity separately. Dots represent quadratic means adjusted for age and sex and error bars represent 95% confidence intervals. Plasma triglycerides and plasma cholesterol were measured on fresh blood samples using routine hospital assays. Triglyceride and cholesterol content in lipoprotein fractions were measured using nuclear magnetic resonance spectroscopy and corrected for recovery. The y-axes are on a logarithmic scale. Bonferroni corrected *p*-values based on 8 parallel tests using unpaired Student *t*-test versus fasting levels (≥8 hours) are as follows: *p<0.05; **p<0.01; ***p<0.001. Abbreviations:HDL: high-density lipoproteins. IDL: intermediate-density lipoproteins. LDL: low-density lipoproteins. VLDL: very low-density lipoproteins.

In analyses stratified according to BMI, individuals were divided into two groups based on the WHO classification of overweight (BMI≥25kg/m^2^); one group with BMI <25 kg/m^2^ and another group with BMI≥25kg/m^2^ (Figure 4). Lipid responses following meals were similar in the two BMI groups; however, in general, individuals with BMI≥25kg/m^2^ had higher levels of plasma triglycerides, VLDL triglycerides, VLDL cholesterol, and VLDL particle number, and lower level of HDL cholesterol.

## Discussion

In this cross-sectional study of 25,656 individuals from the Copenhagen General Population Study selected for metabolomic profiling, we found that triglyceride content increased while cholesterol content decreased in HDL and LDL+IDL fractions following normal meals in individuals in the general population. These findings are novel.

Mechanistically, these finding can be explained in a simply and straight forward manner. Our results are likely related to the close interaction between HDL, LDL, and triglyceride-rich remnant lipoproteins in their metabolic pathways, which is in part regulated by the action of the cholesteryl ester transfer protein (CETP)^25,26^. This enzyme facilitates the bidirectional transfer of cholesteryl esters for triglycerides from HDL and cholesterol-rich LDL to triglyceride-rich remnant lipoproteins and triglyceride-rich LDL^26–29^. Accordingly, even though triglycerides are mainly carried by triglyceride-rich remnant lipoproteins, postprandially, HDL and LDL appear to be enriched with triglycerides likely due to the action of CETP.

Food intake is important when evaluating high plasma triglycerides, and previous epidemiological studies have reported that post-prandial hypertriglyceridemia is an independent risk factor for cardiovascular events^5–7^. Nevertheless, to our knowledge, this is the first population-based study to investigate the triglyceride and cholesterol content of lipoprotein fractions following normal meals in individuals in the general population. In support of our findings, studies on postprandial lipid response to oral fat tolerance tests in short-term studies of selected groups of individuals or patients have likewise found that triglyceride content increased while cholesterol content decreased in HDL and LDL+IDL fractions^14–16^. In contrast, one study^13^ reported decreased LDL triglycerides and another study^30^ reported decreased triglyceride content and increased cholesterol content in HDL and LDL fractions following oral fat tolerance tests; however, only 22 and 8 healthy individuals were included in the two studies, and therefore, their findings may be different from the overall pattern in the general population. Also, in a long-term population-based study, low high-density lipoprotein (HDL) cholesterol was suggested as a stable marker of elevated plasma triglycerides and triglyceride-rich remnant lipoproteins, like high HbA1c is a stable marker of elevated plasma glucose^18^; this is in line with the only minimal postprandially changes in HDL cholesterol observed in the present study.

Strengths of the present study include the large sample size representing the Danish adult general population with direct assessment of lipids including triglyceride and cholesterol content of 14 lipoprotein fractions available. Another strength is the fact that time since last meal was recorded by the examiner immediately before blood sampling, simply by a direct question to the participant at this time.

Possible limitations include that total plasma triglycerides and plasma cholesterol measured by NMR spectroscopy were lower than reported by standard hospital assays. To account for this, cholesterol and triglyceride content in the various lipoprotein fractions were corrected for recovery relative to total plasma cholesterol and plasma triglycerides measured by standardized biochemical assays as done in previous studies^22–24^. This means that the values presented in the present study are directly relatable to values from normal lipid profiles, which could be perceived as a strength. Another potential limitation is that samples were stored at -80 °C before NMR measurements, which could have influenced the composition of lipoprotein fractions; however, we are not aware of any data suggesting this is an issue. Finally, all participants were white people of Danish descent, and accordingly, our findings may not generalize to other ethnic groups, although we are not aware of any data to suggest that these results may not apply to other ethnicities.

Clinically, our findings suggest that triglyceride content increases while cholesterol content decreases in HDL and IDL+LDL fractions following normal meals, and thus indirectly endorse use of non-fasting lipid measurements for risk prediction in the general population as non-fasting measurements better capture the average lipid levels in a person, since the non-fasting state dominates during most of a 24-hour cycle^1^. Further, non-fasting lipid profile sampling are practically easier for patients, laboratories, and doctors alike^1,4^. Interestingly, elevated LDL triglycerides are robustly associated with increased risk of atherosclerotic cardiovascular disease^31^, implying that masking of elevated LDL triglycerides by using fasting lipid profiles makes it more difficult for the physician to evaluate the accurate risk profile in a patient.

In conclusion, in this study of 25,656 individuals from the general population, we observed that triglyceride content increased while cholesterol content decreased in HDL and LDL+IDL fractions following normal meals.

## Supplementary Material

Abstract

## Figures and Tables

**Table 1:** Baseline characteristics of 25,656 individuals in the Copenhagen General Population Study according to time since the last meal.

		Time since last meal, hours								
	All n=25,656	Fasting n=377	0–1 n=3,113	1 –2 n=4,742	2–3 n=6,771	3–4 n=5,544	4–5 n=3,051	5–6 n=1,398	6–7 n=432	7–8 n=228
Women	13,543 (53%)	148 (39%)	1,831 (59%)	2,599 (55%)	3,743 (55%)	2,938 (53%)	1,418 (46%)	575 (41%)	190 (44%)	101 (44%)
Age, years	61(50–72)	58 (49–68)	55 (47–65)	59 (49–69)	63 (53–73)	64 (53–74)	59(50–71)	57 (48–67)	60 (50–72)	64 (52–74)
Albumin, μmol/L	605 (566–645)	628(588–664)	603 (566–643)	599 (562–638)	598 (560–636)	602 (564–643)	618 (578–659)	631 (590–671)	620 (586–666)	617 (579–659)
BMI, kg/m^2^	26 (23–29)	26 (24–29)	26 (23–28)	26 (23–29)	26 (23–29)	26 (23–29)	26 (24–29)	26 (23–29)	26 (23–29)	27 (24–30)

Data are n (%) for categorical variables and median (interquartile range) for continuous variables. Baseline characteristics were obtained at the day of examination. Time since last meal (hours) was recorded by the examiner immediately before blood sampling. Time since last meal was obtained once for each participant. BMI = body mass index.
