# Supplementary material for: Triglyceride content increases while cholesterol content decreases in HDL and LDL+IDL fractions following normal meals: the Copenhagen General Population Study of 25,656 individuals
Source: Atherosclerosis. Author manuscript; Available in PMC 2024 Jan 4. (PMC7615473; doi:10.1016/j.atherosclerosis.2023.117316)
Supplement: Abstract [file EMS192936-supplement-Abstract.pptx]

## Slide 1
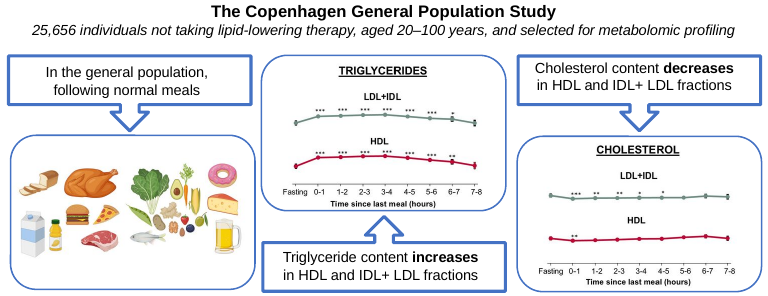

The Copenhagen General Population Study
25,656 individuals not taking lipid-lowering therapy, aged 20–100 years, and selected for metabolomic profiling
Cholesterol content decreases in HDL and IDL+ LDL fractions
In the general population, following normal meals
Triglyceride content increases in HDL and IDL+ LDL fractions
